# Effects of Recovery Time during Magnetic Nanofluid Hyperthermia on the Induction Behavior and Efficiency of Heat Shock Proteins 72

**DOI:** 10.1038/s41598-017-14348-2

**Published:** 2017-10-24

**Authors:** Jung-tak Jang, Jin Wook Jeoung, Joo Hyun Park, Won June Lee, Yu Jeong Kim, Jiyun Seon, Minkyu Kim, Jooyoung Lee, Sun Ha Paek, Ki Ho Park, Seongtae Bae

**Affiliations:** 1 Nanobiomagnetics and Bioelectronics Laboratory (NB2L), Department of Electrical Engineering, University of South Carolina, Columbia, SC 29208 USA; 2 Department of Ophthalmology, Seoul National University College of Medicine, Seoul, 110-744 Republic of Korea; 3 Biomedical Research Institute, Cancer Research Institute, and Ischemic/Hypoxic Disease Institute, Department of Neurosurgery, Seoul National University College of Medicine, Seoul, 110-744 Republic of Korea; 4 Department of Neurosurgery, Seoul National University College of Medicine, Seoul, 110-744 Republic of Korea

## Abstract

In this study, we investigated the effects of recovery time during magnetic nanofluid hyperthermia (MNFH) on the cell death rate and the heat shock proteins 72 (HSP72) induction behavior in retinal ganglion cells (RGCs-5) to provide a possible solution for highly efficient ocular neuroprotection. The recovery time and the heat duration time during MNFH were systematically controlled by changing the duty cycle of alternating current (AC) magnetic field during MNFH. It was clearly observed that the cell death rate and the HSP72 induction rate had a strong dependence on the recovery time and the optimizated recovery time resulted in maximizing the induction efficiency of HSP72. Controlling the recovery time during MNFH affects not only the cell death rate but also HSP72 induction rate. The cell death rate after MNFH was dramatically decreased by increasing the recovery time during MNFH. However, it was also found that the HSP72 induction rate was slightly decreased by increasing the recovery time. These results indicate that applying the appropriate or optimized recovery time during MNFH can improve the induction efficiency of HSP72 by minimizing the cell death caused by cytotoxic effects of heat.

## Introduction

Induction of “heat shock proteins (HSPs)”, particularly HSP70 or HSP72 families, in retinal ganglion cells (RGCs) or optic nerve head has been considered to be a potential clinical modality for ocular neuroprotection in glaucoma since its physiological effectiveness has been demonstrated from the damaged rat eye^1–5^. Hyperthermia as well as various other stress conditions, such as hypoxia, infection, medication, and exposure of cells to ultraviolet light and trace metals, can induce HSPs, which are mediated by activation of nuclear so-called “heat shock factors (HSF)”^6–8^. Among the several stress conditions, HSPs induction *via* hyperthermia treatment has been paid considerable attention in the clinics because hyperthermia is expected to minimize the chemical or biological “side effects” and “toxic problems”^9–11^. For instance, some hyperthermia treatment methods such as whole body hyperthermia using a warming blanket and local hyperthermia using a laser diode or magnetic nanoparticles were employed to induce HSPs in both *in-vivo* and *in-vitro* environments, and their effectiveness in the induction of HSPs was successfully demonstrated^6,12–18^. In order to achieve a highly efficient ocular neuroprotection in glaucoma, HSPs should be induced with a high induction rate as well as a low cell death rate in RGCs. However, although the above different hyperthermia treatment methods successfully induced HSPs, a low induction efficiency of HSPs due to a high cell death rate caused by the cytotoxic effects of heat on the cells was revealed as a critical challenge for future clinical applications. It was reported that the cells undergo a “rapid mode of cell death” when they are exposed to heat (hyperthermia or heating environments)^19^. In addition, it was observed that hyperthermia can induce microscopically detectable damage to the mitotic apparatus^6,19,20^. This damage results in inefficient mitosis and consecutive polyploidy. Fortunately, however, the cytotoxic effects of hyperthermia on the cells have been shown to be biologically related with the exposure time (heat duration time) and intensity of the heat. Therefore, it is expected that the cell death rate can be minimized by controlling the intensity of the heat and its duration time in cells during hyperthermia, thereby improving the induction efficiency of HSPs.

**Figure 1:**
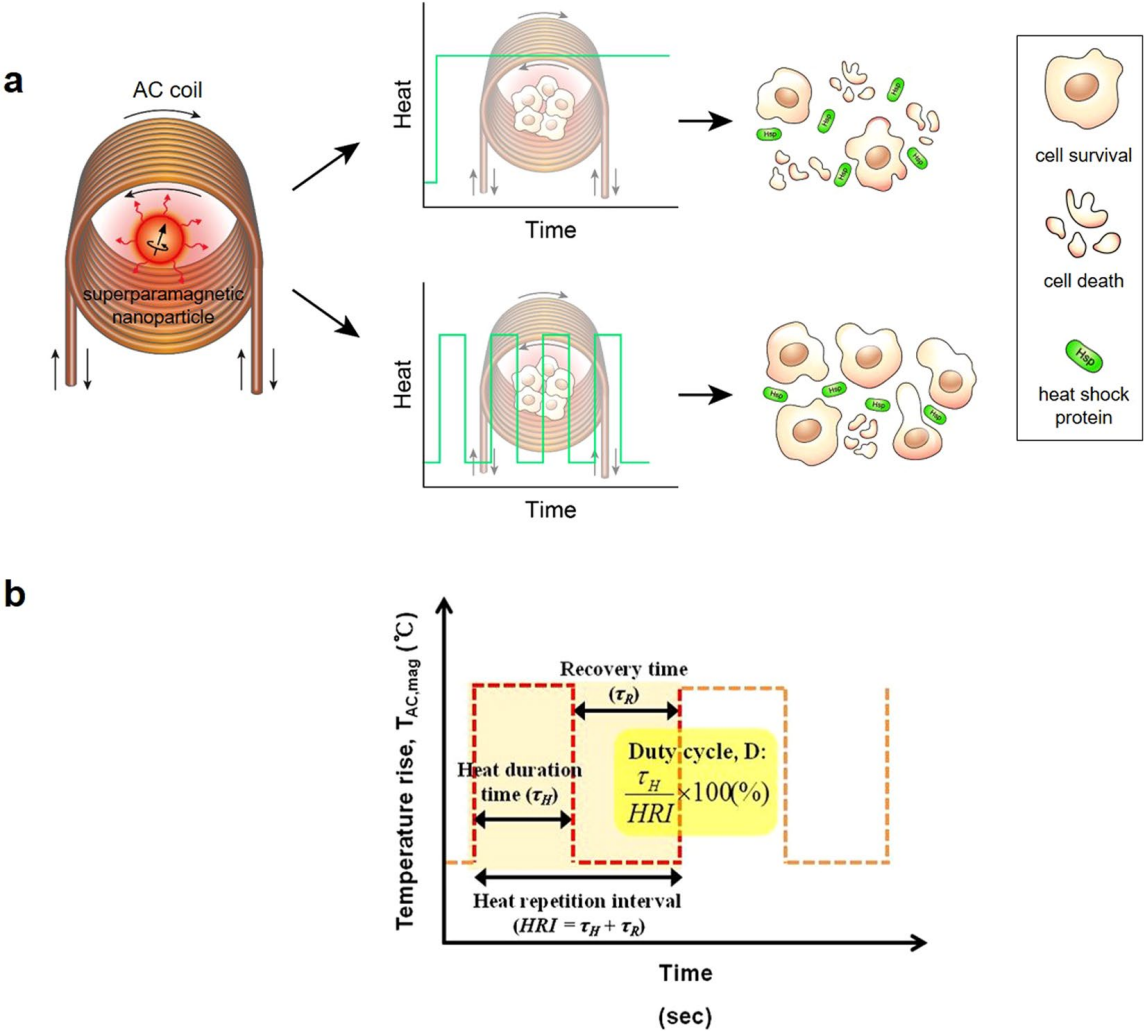
Illustration of HSPs induction characteristics by MNFH with different AC magnetic wave forms. (**a**) A schematic diagram describing the increasing recovery time (τ_R_) can improve the cell survival rate. (**b**) A schematic diagram of the AC magnetic field with duty cycle during MNFH. The D, duty cycle, is defined as the ratio of the τ_H_ to the heat repetition interval (HRI = τ_H_ + τ_R_).

In this study, we systematically controlled the ratio of the recovery time (τ_R_) to the heat duration time (τ_H_) of AC magnetic field (or AC heating stress) during magnetic hyperthermia to explore the effects of recovery time on the induction efficiency behavior of HSP72 in RGCs-5 for ocular neuroprotection in future glaucoma clinics. Magnetic nanofluid hyperthermia (MNFH) with tailored Mn_0.5_Zn_0.5_Fe_2_O_4_ (T-Mn_0.5_Zn_0.5_Fe_2_O_4_) superparamagnetic nanoparticles (SPNPs), which have a good electrical field absorption and a large power loss (200–500 W/m^3^) at a low frequency (<100 KHz)^21^, was employed as a hyperthermia modality to effectively control the τ_R_ and τ_H_ of the AC magnetic field during the HSPs induction process in RGCs-5 (Fig. 1a). In order to control the ratio of τ_R_ to τ_H_ of the AC magnetic field, the duty cycle (D) value of the applied AC magnetic field was systematically varied from 0.25 (25%) to 1 (100%) during MNFH (Fig. 1b). The induction behavior of HSP72 including the induction rate, the induction efficiency, and the cell death characteristics during HSPs induction process were studied by changing the τ_R_ during MNFH. The optimal D value (or the ratio of the τ_R_ to the τ_H_) to provide highly efficient induction of HSP72 in RGCs-5 was empirically investigated.

## Results and Discussion

**Figure 2:**
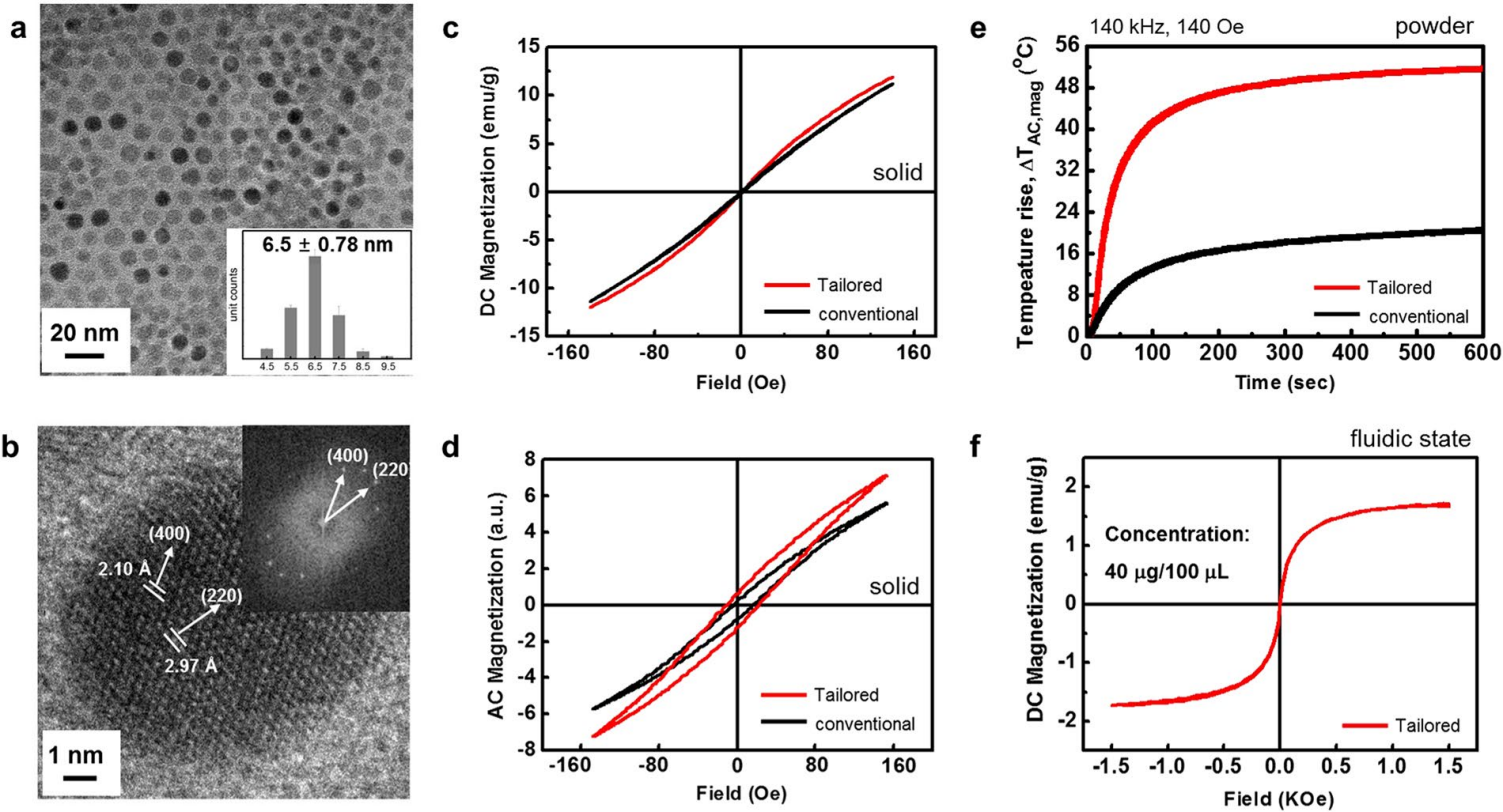
TEM images and magnetic properties of T-Mn_0.5_Zn_0.5_Fe_2_O_4_ and C-Mn_0.5_Zn_0.5_Fe_2_O_4_ nanoparticles. (**a**) Low and (**b**) high magnetification of TEM image for T-Mn_0.5_Zn_0.5_Fe_2_O_4_ nanoparticles. The inset shows the pattern of fast Fourier Transformation (FFT). (**c**) Direct Current (DC) minor hysteresis loops measured at a sweeping field of H_appl_ = ±140 Oe. (**d**) Alternating Current (AC) hysteresis loops measured at a f_appl_ = 140 kHz and H_appl_ = ±140 Oe. (**e**) AC heating chcracteristics measured at a f_appl_ = 140 kHz and H_appl_ = ±140 Oe of T-Mn_0.5_Zn_0.5_Fe_2_O_4_ and C-Mn_0.5_Zn_0.5_Fe_2_O_4_ nanoparticles at powder state. (**f**) A major hysteresis loop of fluidic T-Mn_0.5_Zn_0.5_Fe_2_O_4_@PEG nanoparticles measured at the sweeping field of ±1.5 kOe.

T-Mn_0.5_Zn_0.5_Fe_2_O_4_ nanoparticles were prepared using a modified one-pot thermal decomposition method, which includes metal precursors and reductant in the presence of surfactants and solvent. As can be clearly seen in Fig. 2a, the synthesized T-Mn_0.5_Zn_0.5_Fe_2_O_4_ nanoparticles had a spherical (round) shape with a mean particle (core) size of 6.5 nm ± 0.78 nm. In addition, as shown in Fig. 2b, high-resolution transmission electron microscopy (HR-TEM) analysis confirmed that the synthesized T-Mn_0.5_Zn_0.5_Fe_2_O_4_ nanoparticles have a highly oriented typical cubic spinel structure with (400) and (220) preferred lattice planes. Crystal structure was determined using an X-ray diffractometer (XRD), and doping level of Mn^2+^ or Zn^2+^ ions was qunatititively analyzed by using an energy dispersive X-ray spectroscopy (EDS) and an inductively coupled plasma atomic emission spectroscopy (ICP-AES) (Spplementary Information Fig. S1). Conventional Mn_0.5_Zn_0.5_Fe_2_O_4_ (C-Mn_0.5_Zn_0.5_Fe_2_O_4_) nanoparticles were also explored for comparison. C-Mn_0.5_Zn_0.5_Fe_2_O_4_ nanoparticles obtained using a conventional experimental method showed similar particle size, size distribution and particle shape to those of T-Mn_0.5_Zn_0.5_Fe_2_O_4_ nanoparticles (Spplementary Information Fig. S2). Figure 2c shows the direct current (DC) minor hysteresis loops of as-synthesized T-Mn_0.5_Zn_0.5_Fe_2_O_4_ and C-Mn_0.5_Zn_0.5_Fe_2_O_4_ nanoparticles (solid state) measured at a sweeping field of H_appl_ =  ± 140 Oe. The two nanoparticles did not exhibit any DC minor hysteresis indicating that the synthesized T-Mn_0.5_Zn_0.5_Fe_2_O_4_ and C-Mn_0.5_Zn_0.5_Fe_2_O_4_ nanoparticles have typical superparamagnetic characteristics. However, as can be seen in Fig. 2d, a larger AC hysteresis loss was observed from the T-Mn_0.5_Zn_0.5_Fe_2_O_4_ nanoparticles. This result indicates that T-Mn_0.5_Zn_0.5_Fe_2_O_4_ nanoparticles are magnetically softer than that of C-Mn_0.5_Zn_0.5_Fe_2_O_4_ nanoparticles under AC magnetic field. Furthermore, it implies that T-Mn_0.5_Zn_0.5_Fe_2_O_4_ nanoparticles would generate a higher AC magnetically-induced heating temperature since the larger AC hysteresis loss area of those indicating that it has a higher AC softness, which is directly related to the larger “Néel relaxation loss power”^22^. Figure 2e shows the AC magnetically-induced heating temperature (T_AC,mag_) of T-Mn_0.5_Zn_0.5_Fe_2_O_4_ and C-Mn_0.5_Zn_0.5_Fe_2_O_4_ in solid state. As we expected from Fig. 2d, the T-Mn_0.5_Zn_0.5_Fe_2_O_4_ had much higher ΔT_AC,mag_ compared to that of C-Mn_0.5_Zn_0.5_Fe_2_O_4_. Figure 2f shows the measured DC hysteresis loop of T-Mn_0.5_Zn_0.5_Fe_2_O_4_@PEG (40 μg/100 μL D.I. water) nanofluids. The observed high mono-dispersity of T-Mn_0.5_Zn_0.5_Fe_2_O_4_@PEG nanofluids can be confirmed from the DC magnetic hysteresis loop, because agglomerated nanoparticles in nanofluids can generally cause undesirable changes in dipole-dipole interactions or degradation of magnetic properties directly resulting in asymmetric hysteresis behaviors^22,23^. The measured hysteresis was symmetric and severe degradation was not observed. No severe magnetic degradation or no change of magnetic intrinsic properties compared to those of solid state indicating that the PEG coated T-Mn_0.5_Zn_0.5_Fe_2_O_4_ SPNPs are well dispersed in the nanofluid and form chemically stable colloidal suspension. Furthermore, this well controlled coating status of T-Mn_0.5_Zn_0.5_Fe_2_O_4_@PEG SPNPs is expected to improve the efficiency of the cellular uptake and the cell viability associated with cell cytotoxicity^24–26^.

**Figure 3:**
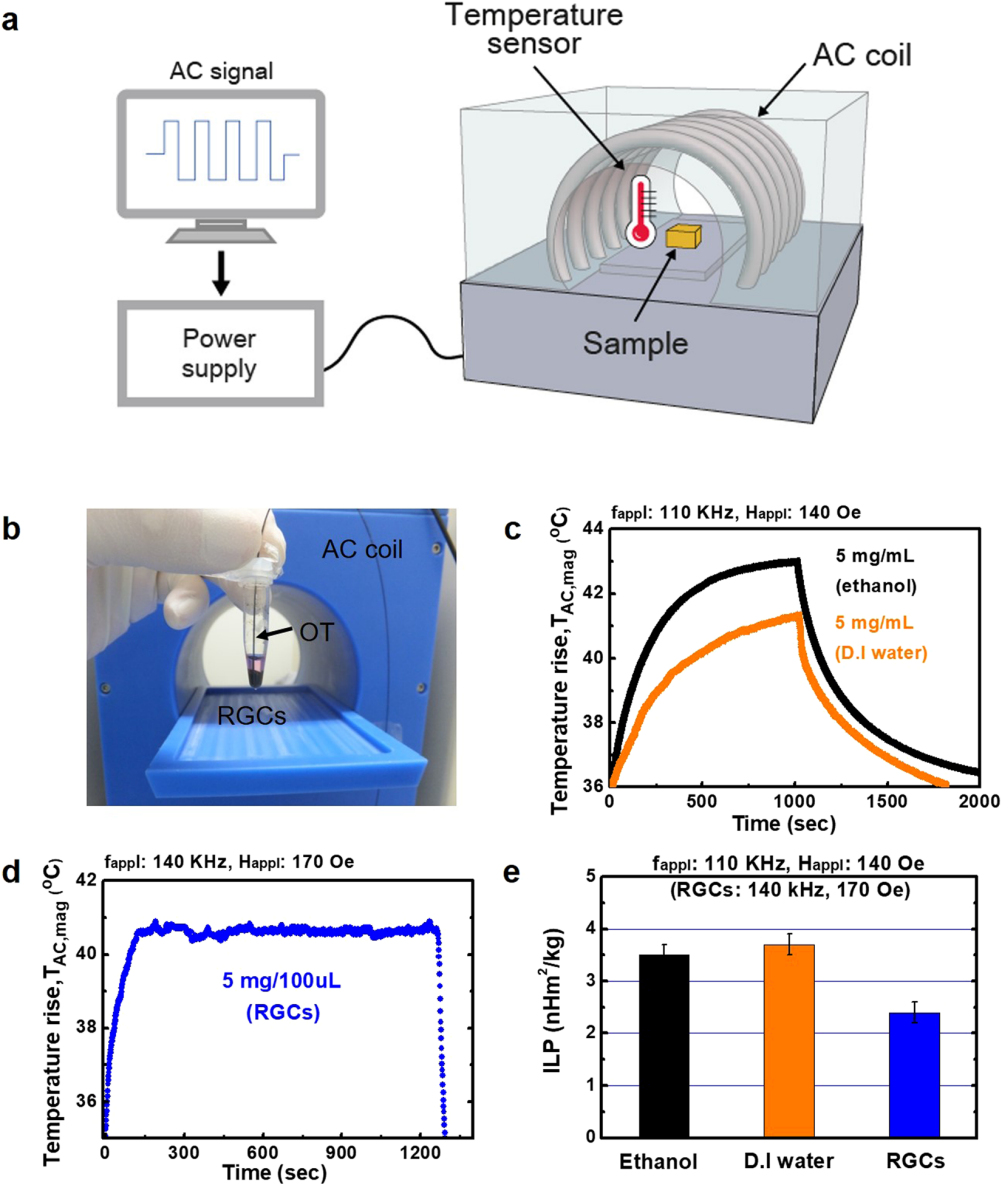
*In vitro* MNFH studies using T-Mn_0.5_Zn_0.5_Fe_2_O_4_@PEG nanofluid for RGCs-5 cells. (**a**) An illustration of the *in vitro* experimental set-up. (**b**) A microcentrifuge tube containing RGCs-5 treated with T-Mn_0.5_Zn_0.5_Fe_2_O_4_@PEG nanofluid with an inserted optical thermometer (OT) for measuring the T_AC,mag_ during HSP72 induction. (**c**) AC magnetically-induced heating characteristics of T-Mn_0.5_Zn_0.5_Fe_2_O_4_@PEG nanofluid measured in ethanol and D.I. water. The f_appl_, and H_appl_ were 110 kHz, and 140 Oe, respectively, and the concentration was 5 mg/mL. (**d**) AC magnetically-induced heating characteristics of RGCs-5 treated with a 500 μg/mL of T-Mn_0.5_Zn_0.5_Fe_2_O_4_@PEG nanofluid. The f_appl_ and H_appl_ were 140 kHz, and 170 Oe, respectively. (**e**) ILP values of T-Mn_0.5_Zn_0.5_Fe_2_O_4_@PEG nanofluids calculated based on the T_AC,mag_ (**c**) and (**d**).

Prior to controlling D value of the AC magnetic field during MNFH with RGCs-5 to study the effects of recovery time on the rate of cell death and the corresponding efficiency of the induction of HSP72, the T_AC,mag_ and intrinsic loss power (ILP) of T-Mn_0.5_Zn_0.5_Fe_2_O_4_@PEG SPNPs were measured in three different media (ethanol, D.I. water, and RGCs-5 cells) to explore AC self-heating characteristics of T-Mn_0.5_Zn_0.5_Fe_2_O_4_@PEG SPNPs under different microenvironments. For this measurement, the micro-centrifuge tube containing the T-Mn_0.5_Zn_0.5_Fe_2_O_4_@PEG SPNPs with ethanol, D.I. water, and RGCs-5, was placed at the center of AC magnetic coil (Fig. 3a and b). The concentration of the T-Mn_0.5_Zn_0.5_Fe_2_O_4_@PEG nanofluid was a 5 mg/mL and the applied f_appl_, and H_appl_ were fixed at a 110 kHz, and a 140 Oe, respectively. After the power is turned on, the T_AC,mag_ of T-Mn_0.5_Zn_0.5_Fe_2_O_4_@PEG nanofluids was rapidly increased and saturated at a 43 °C (in ethanol) and a 41.3 °C °C (in D.I. water) within 1000 sec. with different AC heating up rate due to the different specific heat capacities and viscosities of the two nanofluids (Fig. 3c). In addition, after the AC magnetic field was removed, T_AC,mag_ was decreased sharply for both two nanofluids. To measure the T_AC,mag_ of T-Mn_0.5_Zn_0.5_Fe_2_O_4_@PEG SPNPs in RGCs-5, RGCs-5 were incubated with a 5 mg/10 mL (DMEM) of T-Mn_0.5_Zn_0.5_Fe_2_O_4_@PEG nanofluid for 24 hours at 37 °C. After the incubation process, the RGCs-5 uptake with T-Mn_0.5_Zn_0.5_Fe_2_O_4_@PEG SPNPs were centrifuged to form a cell pellet and a 100 μL of DMEM was added to the RGCs-5 pellet. The T_AC,mag_ of RGCs-5 pellet was measured at a fixed AC magnetic field of f_appl_ = 140 kHz and H_appl_ = 170 Oe. The T_AC,mag_ of the RGCs-5 pellet treated with T-Mn_0.5_Zn_0.5_Fe_2_O_4_@PEG SPNPs was increased up to a 40 °C within a 122 sec, and then it was stably saturated at a typical HSPs induction temperature of 40.5 °C ± 0.5 °C by adjusting the intensity of H_appl_ (Fig. 3d). The ILP values of the T-Mn_0.5_Zn_0.5_Fe_2_O_4_@PEG SPNPs obtained in ethanol, D.I. water, and RGCs-5 were a 3.5 nHm^2^kg^−1^, a 3.7 nHm^2^kg^−1^, and a 2.4 nHm^2^kg^−1^, respectively (Fig. 3e). The differences in obtained ILP values of T-Mn_0.5_Zn_0.5_Fe_2_O_4_@PEG SPNPs in different microenvironments (ethanol, D.I. water, and RGCs-5 cells) directly indicates that the contribution of “Néel” and “Brownian” relaxation heating power to the total heating power is dependent on the nanofluidic ambient environment. A higher viscosity resulted in a lower heating power due to the reduction of “Brownian relaxation loss power” caused by the increase of Brownian relaxation time. This indirectly implies that the “Néel relaxation loss power” would be dominant in characterizing the AC heat generation of superparamagnetic nanoparticles in intercellular or *in-vivo* hyperthermia^27,28^. In addition, the higher ILP of T-Mn_0.5_Zn_0.5_Fe_2_O_4_@PEG nanofluid allows us to apply it for a MNFH agent to systematically and readily control the D value (or τ_R_ and τ_H_) to adjust the induction characteristics during MNFH so that the highly efficient induction of HSP72 in RGCs-5 can be expected to be achieved.

**Figure 4:**
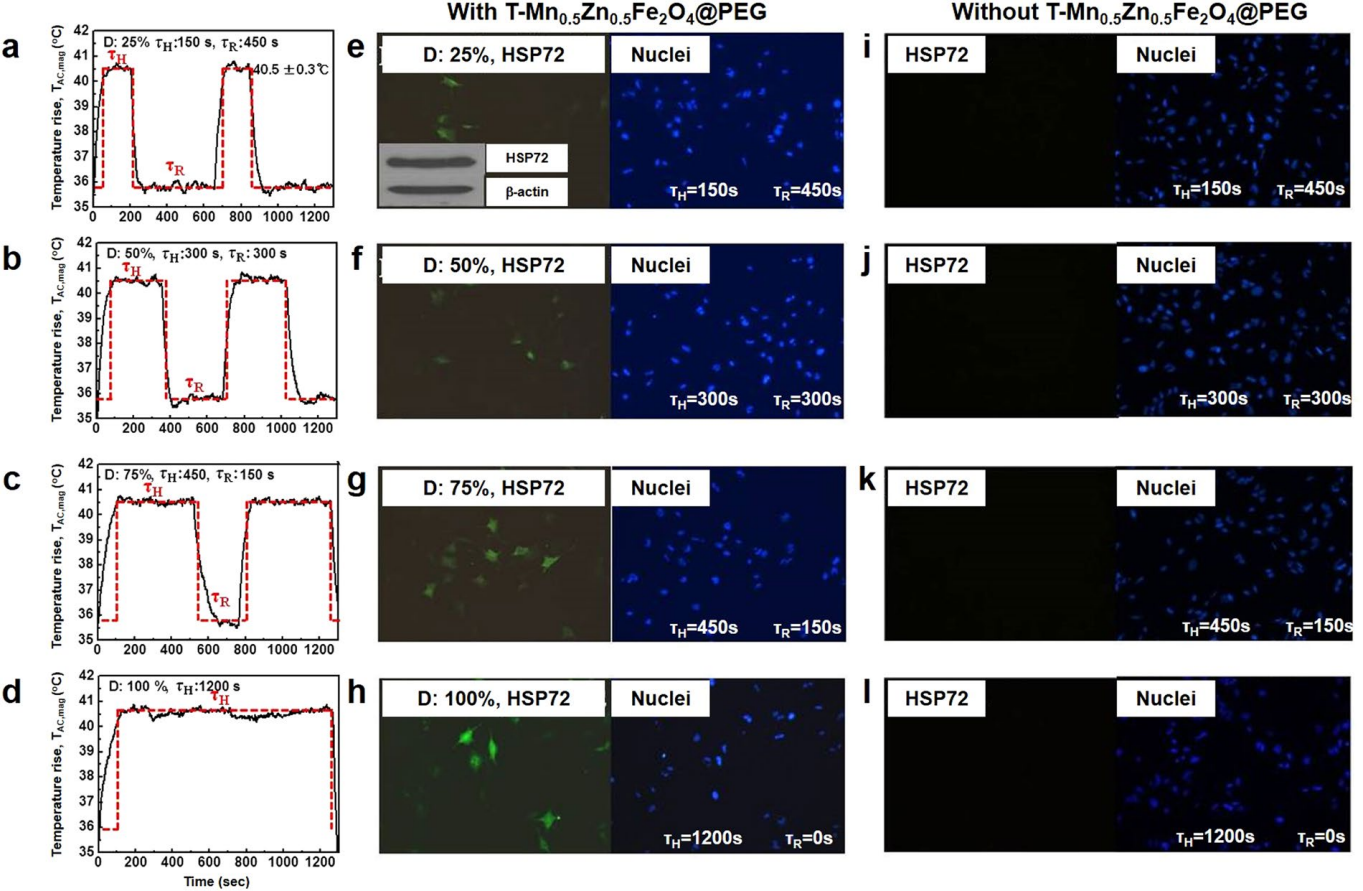
The D value controlled T_AC,mag_ of RGCs-5 treated with a 500 μg/mL of T-Mn_0.5_Zn_0.5_Fe_2_O_4_@PEG nanofluid. Identification of induction of HSP72 (left) after MNFH controlled the D value of the AC magnetic field and (right, control group) after applying only the AC magnetic field (no nanofluid). (**a**) D = 25% (0.25). (**b**) D = 50% (0.5). (**c**) D = 75% (0.75). (**d**) D = 100% (1). ((**e**), inset image) Western blot finding of HSP72 in RGCs-5. (**e**–**h**) the images of stained HSP72 and nuclei for investigating the dependence of HSP72 induction and the cell death rate on the controlling D value of the AC magnetic field treated with T-Mn_0.5_Zn_0.5_Fe_2_O_4_@PEG. (**i**–**l**) the images of stained HSP72 and nuclei without T-Mn_0.5_Zn_0.5_Fe_2_O_4_@PEG.

After confirming the high AC magnetically-induced heating characteristics of T-Mn_0.5_Zn_0.5_Fe_2_O_4_@PEG nanofluid, we conducted systematic studies about the effects of recovery time during MNFH on the characteristics of the cell death and the efficiency of the induction of HSP72 in RGCs-5. Figure 4a–d shows the D value controlled T_AC,mag_ curves (or AC heating stress curves) of RGCs-5 pellet treated with T-Mn_0.5_Zn_0.5_Fe_2_O_4_@PEG nanofluid (500 μg/mL). The D values of the AC magnetic field were systematically changed at a typical HSPs induction temperature of 40.5 °C ± 0.5 °C by adjusting the H_appl_ (τ_H_: 170 Oe (40.5 °C ± 0.5 °C) and τ_R_: 100 Oe (~36 °C)) at the fixed f_appl_ of 140 kHz. The HRI time in one cycle of the AC magnetic field was 600 sec and thus the corresponding τ_R_ values were 450 sec (D: 25%, τ_H_: 150 sec), 300 sec (D: 50%, τ_H_: 300 sec), 150 sec (D: 75%, τ_H_: 450 sec), and 0 sec (D: 100%, τ_H_: 1200 sec).

After MNFH was carried out at the different conditions of D values of AC magnetic field in RGCs-5, the Western blot analysis was carried out to identify the induction of HSP72 and subsequently, the dependence of HSP72 induction. In addition, the cell death rate depending on the D values of AC magnetic field was investigated by using optical microscope images of the stained RGCs-5. The inset image in Fig. 4e shows the Western blot result of the RGCs-5 after MNFH. As can be seen in the Figure, a strong immunoreactivity for HSP72 was detected. Figure 4e–h show the images for the stained HSP72 and nuclei of the RGCs-5 treated with MNFH using T-Mn_0.5_Zn_0.5_Fe_2_O_4_@PEG nanofluid (experimental group, left) and the RGCs treated with only AC magnetic field but no T-Mn_0.5_Zn_0.5_Fe_2_O_4_@PEG (control group, right). The right images (Fig. 4i–l) apparently show that the HSP72 were not induced in the control group. However, the images on the left side clearly demonstrate that the HSP72 were successfully induced by MNFH. The HSP72 induction rate (defined by “visible number of HSP72”) was monotonically increased by increasing the D value of applied AC magnetic field, but in contrast, it was also observed from the microscopic images that the cell survival rate (defined by “visible number of cells or nuclei”) was gradually decreased by increasing the D value of applied AC magnetic field (or AC heating stress). These results clearly illustrate that the change of D value during MNFH directly influence on the extent of the induction of HSP72 as well as the cell death behavior in RGCs-5 during MNFH induced heat shock protein process. In order to numerically analyze the effects of controlling D value of the applied magnetic field on the induction efficiency of HSP72, the relative numerical calculation methods were employed for quantitative analysis. First, the HSP72 induction rate (denoted by “R_H_” = (visible number of HSP72/visible number of nuclei) × 100 (%)) and the cell (nucleus) death rate (denoted by “R_C_” = 1 – (visible number of nuclei after MNFH / visible number of nuclei in the control group) × 100 (%)) were calculated using the optical microscopic images of stained RGCs-5 by considering for both experimental and control groups, and accordingly, the induction efficiency, η, of HSP72 was obtained from the analyzed R_H_ and R_C_, as shown in equation (1).

1 $${\eta }=\frac{{{R}}_{{H}}}{{{R}}_{{C}}}\times 100( \% )$$

Table 1 shows the analyzed results of R_H_ and R_C_ in RGCs-5 after MNFH in which the D value of the applied AC magnetic field was changed from 0.25 (25%) to 1 (100%). According to the analyzed results, the D value of 25% (duty factor: 0.25) exhibited the lowest R_H_ of 10.3% and the R_C_ of 22.1%, while the D value of 100% (duty factor: 1) had the highest R_H_ of 26.9% and the R_C_ of 65.4%. In addition, Table 1 shows that the both R_C_ and R_H_ were decreased by increasing the τ_R_. (In other words, the R_C_ and R_H_ were proportional to the heat duration time, τ_H_). This result simply indicates that the induction behavior (induction rate and cell death rate) of HSP72 in RGCs-5 has a dependence on the duty cycle of applied AC magnetic field. However, it is interestingly noted from Table 1 that the R_C_ (cell death rate) was more significantly reduced than that of R_H_ by increasing the recovery time (τ_R_). Accordingly, we speculate that the increase of the τ_R_ during MNFH can contribute to improve the cell survival rate by allowing the cells to have the time to recover from the applied AC magnetic field. Thus, in order to achieve a high η of HSP72, the ratio of the τ_R_ to the τ_H_ during MNFH in RGCs-5 was tried to be optimized. The η of HSP72 determined by the ratio of the τ_R_ to the τ_H_ (or duty factor) was calculated using equation 1. As shown in Table 1, the optimal ratio of the τ_R_ to the τ_H_ for the highest η of HSP72 induction was found to be a 1: 1 (duty factor D = 0.5 (50%)). This result demonstrates that applying the same period of τ_R_ as the τ_H_ to the AC magnetic field during MNFH can maximize the η of HSP72 induction by reducing the cell death rate, R_C_. It is well known that cells respond to various kinds of stresses in selective ways that help them to either survive the stressful stimuli or signal cell death pathway that can eventually eliminate injured cells. Whether cells mount a protective or detrimental stress response is determined by the cell type and especially, by the nature and the duration of the stress^29,30^. To our knowledge, our study is the first report that has manipulated the duration of applied AC magnetic field (AC magnetic heating stress) by introducing recovery time, during MNFH to induce HSP72 in RGCs-5. Our research on the effects of recovery time during MNFH induced HSP72 in RGCs-5 clearly demonstrates that an appropriate recovery time is required to minimize the death of cells while maximizing the induction rate of HSP72. When the same period of τ_R_ and τ_H_ is applied to the AC magnetic field, it was clear that cells undergo heat absorption time and heat dissipation time recurrently at regular intervals. This could provide a large population of cells not only with adequate heat duration time (τ_H_), but also with enough recovery time (τ_R_) from the stress before entering into cell death pathways, reducing the undesirable side effects of MNFH and eventually leading to the highest η of HSP72. In light of this, this research strongly supports the clinical feasibility of MNFH that induces the localized HSPs for the efficient ocular neuroprotection modality in glaucoma clinics.

**Table 1:** The calculated results of cell death and HSP72 induction rate and induction efficiency of HSP72 after MNFH controlled the D value of the AC magnetic field.

Duty cycle value (D)	Cell death rate (Mean ± SD)	HSP72 induction rate (Mean ± SD)	HSP72 induction efficiency (%)
0.25	22.1 ± 0.8*	10.3 ± 0.5*	46.6
0.5	30.0 ± 1.3*	18.0 ± 0.7*	60.0
0.75	53.0 ± 2.1*	23.0 ± 1.1*	43.4
1.0	65.4 ± 3.2*	26.9 ± 1.3*	41.1

*P-value < 0.05 on student *t*-test.

## Conclusions

We investigted the effects of τ_R_ during MNFH on the induction rate of HSP72 and cell death behavior of RGCs-5 to achieve the most efficient η of HSP72 induction for clinical applications. It was experimentally demonstrated that the controlling τ_R_ during MNFH affects not only the cell death rate but also HSP72 induction rate. The cell death rate after MNFH was dramatically decreased by increasing the τ_R_ during MNFH. However, it was also found that the HSP72 induction rate was slightly decreased by increasing the τ_R_. These results indicate that applying the appropriate or optimized τ_R_ during MNFH can improve the η of HSP72 by minimizing the cell death caused by the cytotoxic effect of the heat. Furthermore, this study is expected to provide a useful solution for the safe and effective induction of HSP72 for the highly efficient ocular neuroprotection for glaucoma.

## Methods

### Synthesis of conventional and magnetically tailored Mn_0.5_Zn_0.5_Fe_2_O_4_ SPNPs and PEG coating for nanofluids

Fe (III) acetylacetonate (precursor, >99.9%), Mn (II) acetate tetrahydrate (99.99%), Zn acetate dihydrate (99.999%), Oleic acid (surfactant, 90%), Oleylamine (surfactant, 70%), 1,2-hexadecanediol (reductant, 90%), and benzyl ether (solvent, 99%) were purchased from Sigma-Aldrich to synthesize Mn_0.5_Zn_0.5_Fe_2_O_4_ SPNPs. In order to coat the nanoparticles with PEG, methoxy-PEG-silane 500 Da, and triethylamine, which were purchased from Gelest Inc., and Sigma-Aldrich, respectively, were used. The conventional Mn_0.5_Zn_0.5_Fe_2_O_4_ (C-Mn_0.5_Zn_0.5_Fe_2_O_4_) SPNPs and magnetically tailored Mn_0.5_Zn_0.5_Fe_2_O_4_ (T-Mn_0.5_Zn_0.5_Fe_2_O_4_) SPNPs were synthesized using a conventional high temperature thermal decomposition (HTTD) method^31^, and a chemically and thermally modified HTTD method, respectively. To synthesize C-Mn_0.5_Zn_0.5_Fe_2_O_4_ SPNPs, Mn (II) (0.5 mmol), Zn (II) (0.5 mmol), Fe (III) (2 mmol), oleic acid (6 mmol), oleylamine (6 mmol), 1.2 hexadecanediol (10 mmol), and benzyl ether (20 mL) were mixed in a three neck flask and magnetically stirred at room temperature. The mixed solutions were heated to 200 °C for 30 min (~6 °C/min, the first ramping up rate) and maintained for another 120 min (nucleation step). Then, the solutions were heated again up to 296 °C, which is a boiling point of benzyl ether, for 8 min (12 °C/min, the second ramping rate) and maintained for 60 min (growth step). While, for the T-Mn_0.5_Zn_0.5_Fe_2_O_4_ SPNPs, the synthesis conditions were chemically and thermally tailored from the conventional HTTD process to control the concentration of cations and their distribution in A (tetrahedral) and B (octahedral)-sites of Fe_3_O_4_. To synthesize the T-Mn_0.5_Zn_0.5_Fe_2_O_4_ SPNPs, Mn (II) (0.5 mmol), Zn (0.5 mmol), Fe (III) (2 mmol), oleic acid (6 mmol), oleylamine (6 mmol), 1.2 hexadecanediol (10 mmol), and benzyl ether (20 mL) were mixed in a three neck flask and magnetically stirred at room temperature. The ramping up rate, and the heat treatment time in the nucleation process (process temperature: ~200 °C) were 11 °C/min, and 60 min, respectively. Then, the reaction solutions were heated again up to 296 °C for 30 min (3.2 °C/min, the second ramping rate) and the heat treatment time in the growth process (process temperature: ~296 °C) was 46.5 min. After the heat treatment process, the mixed solution was cooled down to room temperature and ethanol (40 mL) was added to the mixed solution to rinse the synthesized nanoparticles. The rinsed nanoparticles were collected by centrifugation and then dried at room temperature. The synthesized T-Mn_0.5_Zn_0.5_Fe_2_O_4_ nanoparticles were coated with PEG to form a nanofluid. For coating the PEG layer, the nanoparticles were first dispersed in toluene (7.5 mL), and then PEG (0.75 mL) and triethylamine (catalyst, 3.75 mL) were added to the toluene containing the nanoparticles. The mixed solution was shaken well for 24 hours at room temperature, and then the PEG coated nanoparticles were collected by centrifugation and dispersed in D.I. water.

### Characterization and magnetic properties of C-Mn_0.5_Zn_0.5_Fe_2_O_4_ and T-Mn_0.5_Zn_0.5_Fe_2_O_4_ nanoparticles

The size and morphology of the synthesized C-Mn_0.5_Zn_0.5_Fe_2_O_4_ and T-Mn_0.5_Zn_0.5_Fe_2_O_4_ nanoparticles were analyzed using a transmission electron microscopy (TEM). The DC magnetic hysteresis loops of the uncoated (solid state) and PEG coated (fluid state) nanoparticles were measured using a vibrating sample magnetometer (VSM). In order to measure the magnetic properties of Mn_0.5_Zn_0.5_Fe_2_O_4_@PEG nanofluid, we used a liquid VSM holder (liquid upper and bottom cup) at room temperature. For successful measurement, the liquid sample has to be encapsulated carefully without any air inside the capsules. A methoxy-PEG-silane 500 Da was used as a suitable surfactant to reduce aggregation of nanoparticles in fluidic state.

### AC magnetically-induced heating temperature, T_AC,mag_, and intrinsic loss power, ILP, of T-Mn_0.5_Zn_0.5_Fe_2_O_4_@PEG nanoparticles

The T_AC,mag_ of T-Mn_0.5_Zn_0.5_Fe_2_O_4_@PEG nanofluids was measured in ethanol, D.I. water, and RGCs-5 using an AC magnetic field generation system consisting of AC coils, capacitors, DC power supplies, and wave generators (anytech. Korea). The applied frequency (f_appl_), and H_appl_ were 110 kHz ~ 140 kHz, and 140 Oe ~170 Oe, respectively. The ILP of the T-Mn_0.5_Zn_0.5_Fe_2_O_4_@PEG nanoparticles was determined based on the measured T_AC,mag_ and using equation (2)^32^.

2 $$ILP[nH{m}^{2}k{g}^{-1}]=\frac{SLP\,[Wk{g}^{-1}]}{f[KHz]{H}^{2}[{(KA{m}^{-1})}^{2}]}$$

The SLP was determined based on the measured T_AC,mag_ and using equation (3)^33^.

3 $$SLP[W{g}^{-1}]=\frac{C{V}_{s}}{m}\frac{dT}{dt}$$

(*C*: volumetric specific heat capacity, *V*_*s*_: sample volume, *m*: mass of magnetic material, *dT* and *dt*: the initial slope of the graph of the change in temperature versus time)

### Cell culture, *in-vitro* cytotoxicity assay, and cellular uptake

The rat RGCs-5 were cultured in Dulbecco Modified Eagle Medium (DMEM, Invitrogen, Carlsbad, CA) containing 10% fetal bovine serum (FBS, Introgen), 100 μg/ml penicillin/streptomycin (Invitrogen), and 2 mM glutamine (Invitrogen) in a humidified incubator with 5% CO_2_ at 37 °C. The *in-vitro* cytotoxicity assay of RGCs with T-Mn_0.5_Zn_0.5_Fe_2_O_4_@PEG nanoparticles was conducted at the concentration of 0, 1, 3, 5, 10, 30, 100, 250, and 500 μg/mL using a Cell Counting Kit-8 (CCK-8) assay (Fig. S3). The RGCs-5 cells were exposed to the nanoparticles for one day. For cellular uptake of T-Mn_0.5_Zn_0.5_Fe_2_O_4_@PEG nanoparticles by RGCs-5, the RGCs-5 was differentiated with 1 μM staurosporine for 1 hour and incubated again with a 500 μg/mL of T-Mn_0.5_Zn_0.5_Fe_2_O_4_@PEG nanoparticles in a 10 mL of DMEM for 24 hours. The RGCs-5 containing T-Mn_0.5_Zn_0.5_Fe_2_O_4_@PEG nanoparticles were washed with phosphate buffered saline (PBS) and trypsinized with trypsin-EDTA (Life technologies, USA) followed by addition of 100 μL of DMEM, and then collected in a microcentrifuge tube and centrifuged to form a cell pellet. The volume of the microcentrifuge tube was 0.6 mL. The pellet size was 0.4 × 0.4 cm, when cell preparation was performed in a 0.6 mL tube.

### Control of τ_R_ during MNFH with RGCs-5

An optical thermometer (thermal sensor tip, experimental group) was inserted in the RGCs-5. The thermometer probe tip was precisely located inside the cell pellet (Fig. S5) and were placed at the center of AC coil to apply the AC magnetic field for MNFH. The τ_R_ from AC magnetic field (AC heating stress) during MNFH was changed by controlling the D value of AC magnetic field in RGCs-5. The D value was defined as the ratio of the τ_H_ to the heat repetition interval (HRI = τ_H_ + τ_R_), as shown in Fig. 1(b). The D value was controlled from 25% (0.25) to 100% (1) by tuning the H_appl_ (τ_H_: 170 Oe and τ_R_: 100 Oe) at the fixed f_appl_ of 140 kHz during MNFH. RGCs-5 pellets without T-Mn_0.5_Zn_0.5_Fe_2_O_4_@PEG nanoparticles were also prepared as a control group, and they were undergone the same AC magnetic field conditions as the experimental group done.

### Induction and Identification of HSP72

At 24 hours after MNFH, the RGCs-5 were fixed in 10% formaldehyde for 10 min, and then the fixed RGCs-5 were washed with PBS-20 three times. In order to identify the induction of HSP72 and to study the induction behavior at the intercellular level, the RGCs incubated with nanofluid were collected in a microcentrifuge tube and centrifuged to form a cell pellet. The microcentrifuge tube containing the RGCs pellet was immersed in a hot water bath to make the temperature of RGCs with nanofluids keep in the range of 36 ~ 37 °C. the microcentrifuge tube with an optical thermometer inserted in the RGCs was placed at the center of AC coil to apply AC magnetic field. The heating up rate of “AC magnetically-induced heating stress” of the nanofluids in RGCs was controlled by changing the strength of H_appl_ in the biologically and physiologically safe range of 120 Oe (H_appl_·f = 1.34 × 10^9^ A m^−1^ s^−1^) ~ 160 Oe (H_appl_·f_appl_ = 1.78 × 10^9^ A m^−1^ s^−1^) at the fixed frequency of 140 kHz. The T_AC,mag_ of RGCs with nanofluids during HSPs induction process was kept at the temperature of 40.5 °C ± 0.3 °C for 900 sec. The induction of HSP72 in the RGCs-5 was confirmed by staining the cells. The HSP72 were stained by Anti-HSP72 (primary antibody, Enzo life sciences, USA) and Alexa fluor 488 goat anti-mouse IgG (secondary antibody, FITC, Fluorescein isothiocyanate, Life technologies, USA), and the nucleus were stained by DAPI (4′,6-diamidino-2-phenylindole, Sigma, USA). An Olympus IX51 microscope (OLYMPUS CORPORATION, Tokyo, Japan) was used at 400x magnification for image acquisition. Images were recorded with an DFC500 high-resolution digital camera and processed using LAS (LEICA Application Suite) ver 4.2 software (LEICA Company, Wetzlar, Germany). The induced HSP72 were also identified by employing Western blot analysis. For Western blot analysis, the collected RGCs-5 were lysed using a lysis buffer (Intron Biotechnology, Korea) and loaded onto a 10% SDS-polyacrylamide gel. Subsequently, they were transferred to Polyvinylidene fluoride membrane. The membrane was blocked by non-fat dry milk and incubated with primary antibody (1:1000, Enzo Lifesciences, USA) and ß-actin antibody (1:1000, Sigma, USA). The membrane was washed with a Tris-buffered saline containing 0.1% Tween-20 and incubated in a goat anti-mouse IgG (1:5000, Santa Cruz, USA). The immunoblotting bands were detected by West-zol detection system (Intron Biotechnology, Korea)

### Statistical Analysis

The efficiency and cell death rate data were expressed with mean values along with standard deviations from at least three repetitive experiments. The statistical analysis was done using Prism software 7.0 (GraphPad Software, Inc., San Diego, CA), and student’s t-test were used to compare the data in groups. (*Represents P < 0.05) All the experiments were repeated on separate days, and the data shown in this study are representative of all the repetitions.

## Electronic supplementary material

Supplementary Information
