# Supplementary material for: Effects of Recovery Time during Magnetic Nanofluid Hyperthermia on the Induction Behavior and Efficiency of Heat Shock Proteins 72
Source: Sci Rep. 2017 Oct 24;7:13942. doi: 10.1038/s41598-017-14348-2 (PMC5655350; doi:10.1038/s41598-017-14348-2)
Supplement: Supplementary file 1 — Supplementary Information [file 41598_2017_14348_MOESM1_ESM.doc]

Supplementary Information

Effects of Recovery Time during Magnetic Nanofluid Hyperthermia on the Induction Behavior and Efficiency of Heat Shock Proteins 72

**Jung-tak Jang^1,*^, Jin Wook Jeoung^2,*^, Joo Hyun Park^2^, Won June Lee^2^, Yu Jeong Kim^2^, Jiyun Seon^1^, Minkyu Kim^3^, Jooyoung Lee^3^, Sun Ha Paek^3,4^, Ki Ho Park^2^ and Seongtae Bae^1^**

^1^Nanobiomagnetics and Bioelectronics Laboratory (NB^2^L), Department of Electrical Engineering, University of South Carolina, Columbia, SC 29208, USA

^2^Department of Ophthalmology, Seoul National University College of Medicine, Seoul 110-744, Republic of Korea

^3^Biomedical Research Institute, Cancer Research Institute, and Ischemic/Hypoxic Disease Institute, Department of Neurosurgery, Seoul National University College of Medicine, Seoul, 110-744, Republic of Korea

^4^Department of Neurosurgery, Seoul National University College of Medicine, Seoul, 110-744, Republic of Korea


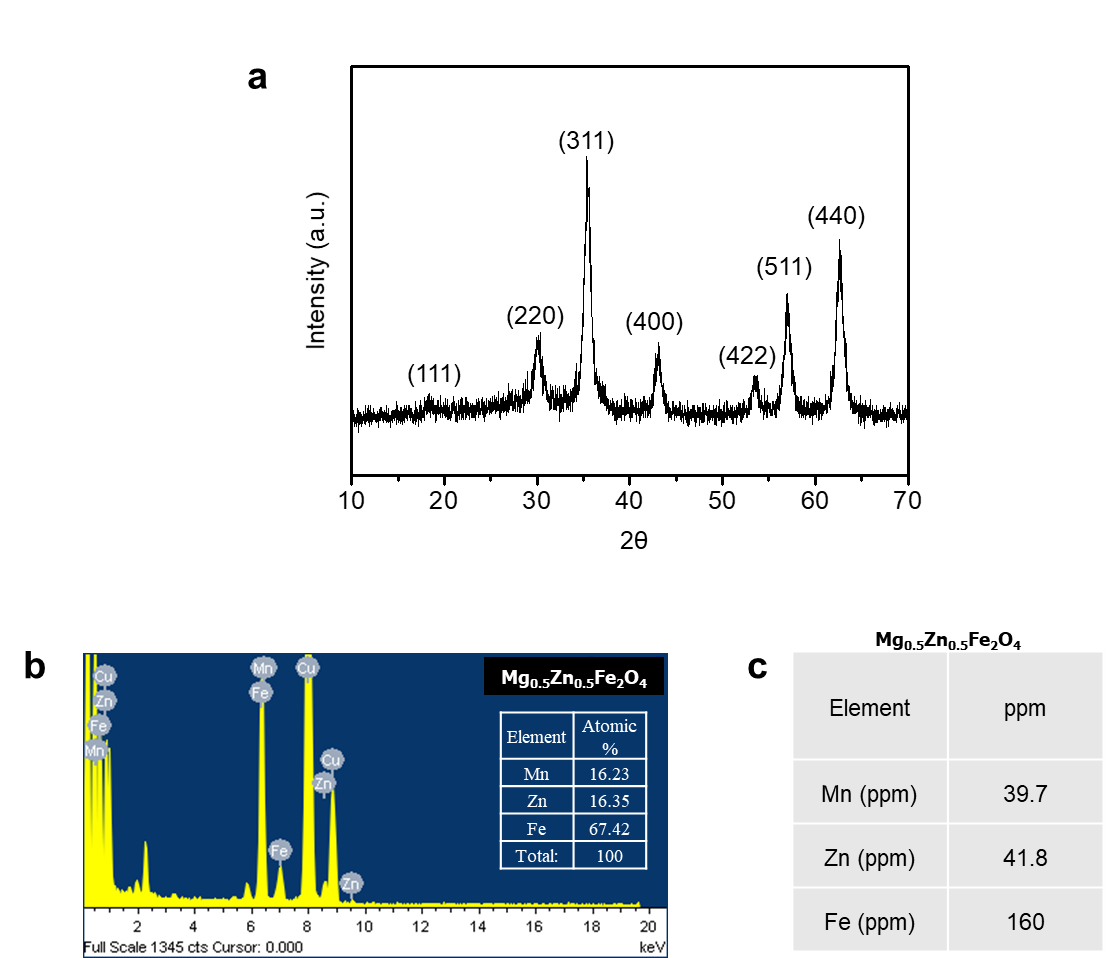


**Figure S1.** **Crystal structure and composition determination of T-Mn_0.5_Zn_0.5_Fe_2_O_4_ nanoparticles.** a) XRD patterens of T-Mn_0.5_Zn_0.5_Fe_2_O_4_ nanoparticles. This patterns are well indexed and correlated to those of a spinel structure. b) EDS data, c) ICP-AES data of T-Mn_0.5_Zn_0.5_Fe_2_O_4_ nanoparticles.


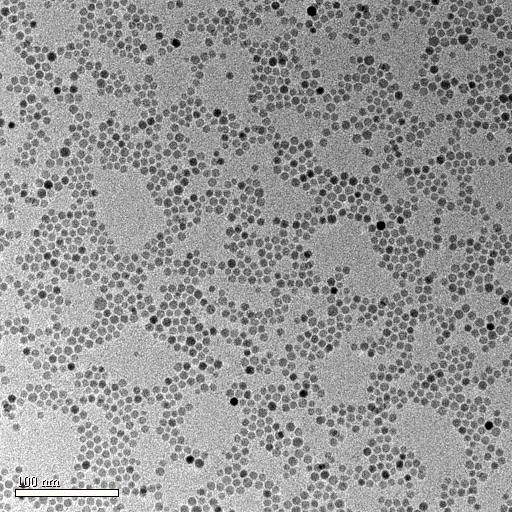


**Figure S2.** **TEM image of as-synthesized C-Mn_0.5_Zn_0.5_Fe_2_O_4_ nanoparticles.**


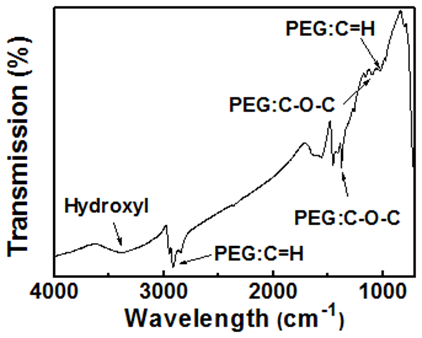


**Figure S3.** **Surface modification of T-Mn_0.5_Zn_0.5_Fe_2_O_4_ nanoparticles.** Fourier transform Infrared (FTIR) spectra of T-Mn_0.5_Zn_0.5_Fe_2_O_4_@PEG nanofluid dispersed in water. For FTIR analysis, the nanofluid was air-dried and mixed with potassium bromide, and then the mixture was pressed into a disc. The C-O-C peaks were appeared at 1103 cm^-1^ and 1344 cm^-1^ in the FTIR spectrum of T-Mn_0.5_Zn_0.5_Fe_2_O_4_@PEG. In addition, C=H stretching vibrations at 955 - 965 cm^-1^ and C=H peak at around 2912 cm^-1^ were appeared, and the broad peak of hydroxyl group was clearly observed in the range of 3400-3550 cm^-1^. This result strongly demonstrates that the surface of T-Mn_0.5_Zn_0.5_Fe_2_O_4_ was successfully modified by PEG layer.


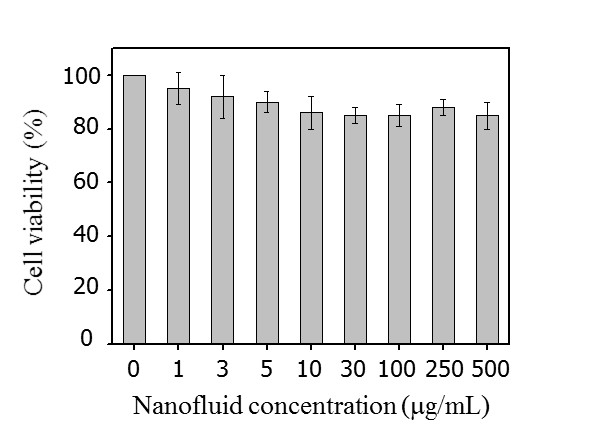


**Figure S4.** **Biocompatibility (Cytotoxicity) of T-Mn_0.5_Zn_0.5_Fe_2_O_4_ nanofluids with RGCs-5 cell line.** For the test, RGCs cell lines were treated with T-Mn_0.5_Zn_0.5_Fe_2_O_4_ nanofluids for one day. They showed a high enough biocompatibility with RGCs-5 cell line even at a higher (500 μg/ml) concentration.


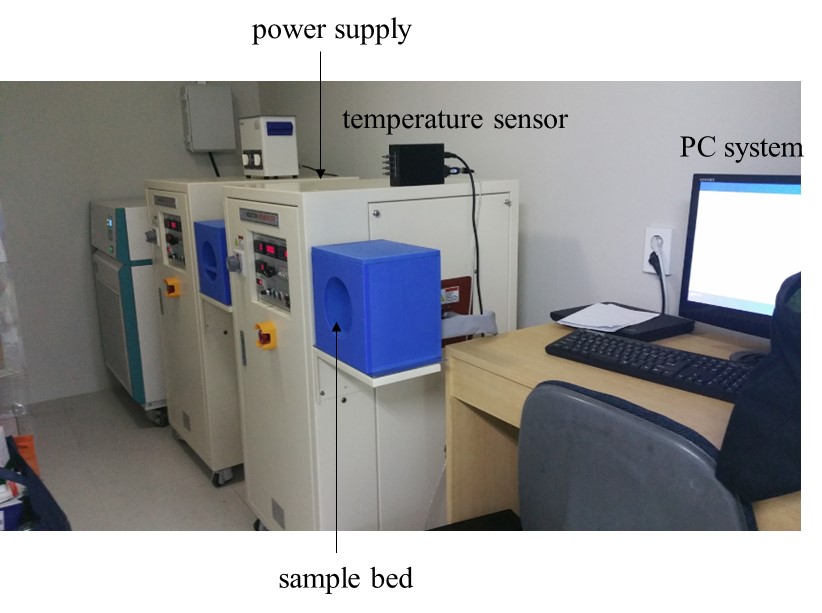


**Figure S5.** **A picture of our newly developed home-made hyperthermia system.** Our system (Model No: ATHF-5) consists of a power supply (output up to 5 KW), a sample bed, an optical temperature sensor (Luxtron® fiber optic thermometry probe), and a desk typed PC.


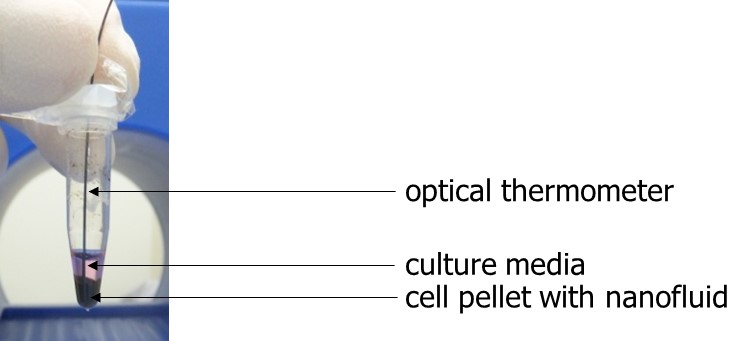


**Figure S6.** **A picture of an ep-tube containing RGCs-5 cell pellet treated with T-Mn_0.5_Zn_0.5_Fe_2_O_4_@PEG nanofluid with an inserted optical thermometer (OT).** The temperature probe tip was precisely located inside the cell pellet and the black component inside the ep-tube is nanofluid with cell pellet.


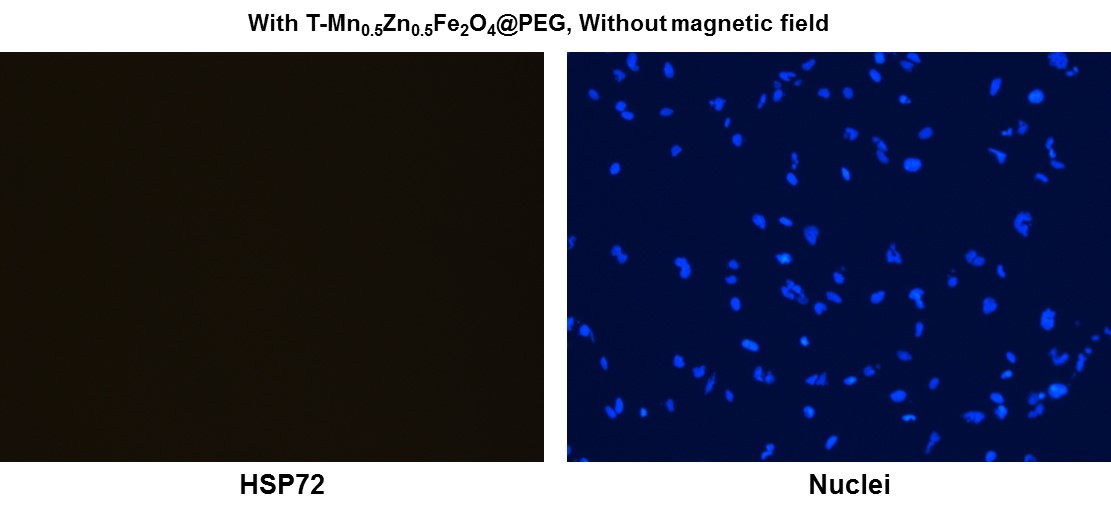


**Figure S7.** **Identification of induction of HSP72 (left) after treating with only nanofluid but no magnetic field.** Figure S7 show the HSP72 was not induced in the RGCs-5 cell after treating with only nanofluid (no magnetic field) until 1200 sec.


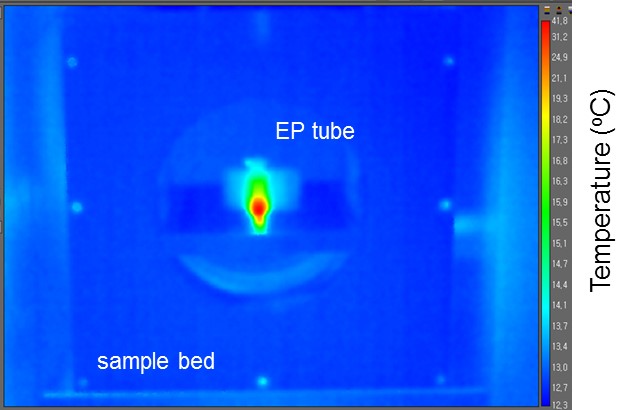


**Figure S8.** Temperature distribution of eppendorf (EP) tube containing RGCs-5 treated with a 500 μg/mL of nanofluids after AC magnetically induced heating. The f_appl_ and H_appl_ were 140 kHz and 170 Oe. The temperature rise of the sample and the surrounding coil was measured by an infrared (IR) camera (FLIR T650sc, Wilsonville , OR, USA) and the thermal images were analyzed by thermography software (FLIR Research IR Max). The distance between the sample and the IR camera was a 50 cm.
